# Vitamin D pathway gene polymorphisms, diet, and risk of postmenopausal breast cancer: a nested case-control study

**DOI:** 10.1186/bcr1642

**Published:** 2007-01-23

**Authors:** Marjorie L McCullough, Victoria L Stevens, William R Diver, Heather S Feigelson, Carmen Rodriguez, Robin M Bostick, Michael J Thun, Eugenia E Calle

**Affiliations:** 1 Epidemiology and Surveillance Research, American Cancer Society, 1599 Clifton Road NE, Atlanta, GA 30329, USA; 2 Department of Epidemiology, Rollins School of Public Health, Emory University, 1518 Clifton Road NE, Atlanta, GA 30322, USA

## Abstract

**Introduction:**

Vitamin D receptor (VDR) polymorphisms have been inconsistently associated with breast cancer risk. Whether risk is influenced by polymorphisms in other vitamin D metabolism genes and whether calcium or vitamin D intake modifies risk by genotype have not been evaluated.

**Methods:**

We conducted a nested case-control study within the Cancer Prevention Study II Nutrition Cohort of associations between breast cancer and four VDR single-nucleotide polymorphisms (SNPs), *Bsm1*,*Apa1*,*Taq1*, and *Fok1*, a poly(A) microsatellite, and associated haplotypes (*baTL *and *BAtS*). We also examined one SNP in the 24-hydroxylase gene (*CYP24A1*) and two in the vitamin D-binding protein (group-specific component [*GC*]) gene. Participants completed a questionnaire on diet and medical history at baseline in 1992. This study includes 500 postmenopausal breast cancer cases and 500 controls matched by age, race/ethnicity, and date of blood collection.

**Results:**

Incident breast cancer was not associated with any genotype examined. However, women with the *Bsm1 bb *SNP who consumed greater than the median intake of total calcium (≥902 mg/day) had lower odds of breast cancer compared to women with the *Bb *or *BB *genotype and less than the median calcium intake (odds ratio 0.61, 95% confidence interval 0.38 to 0.96; *p*_interaction _= 0.01). Similar interactions were observed for *Taq1 *(T allele) and the poly(A) (LL) repeat.

**Conclusion:**

We found no overall association between selected vitamin D pathway genes and postmenopausal breast cancer risk. However, certain VDR gene polymorphisms were associated with lower risk in women consuming high levels of calcium, suggesting that dietary factors may modify associations by VDR genotype.

## Introduction

Vitamin D has been associated with a lower risk of several types of cancer, including breast cancer [1,2]. Its action is mediated through the vitamin D receptor (VDR), a nuclear transcription-regulating factor that signals the synthesis of proteins involved in bone mineral homeostasis and cell-cycle regulation [2]. The receptor is present in most cell types, including normal and neoplastic breast tissue [3]. Clinically, breast tumors with greater VDR expression relapse more slowly after first diagnosis [4].

The VDR gene contains several polymorphisms. Three single-nucleotide polymorphisms (SNPs) located near the 3' region have been examined in relation to breast cancer [2]. These polymorphisms, identified by their restriction endonuclease cleavage sites (*Taq1*,*Bsm1*, and *Apa1*), include an intronic G→A replacement (*Bsm1*, rs1544410, G = *b*), an intronic C→A replacement (*Apa1*, rs7975232, C = *a*), and a T→C replacement in exon 9 (*Taq1*, rs731236, C = *t*), which result in a synonymous change at codon 352. Although not functional, these SNPs are strongly linked with a poly(A) microsatellite repeat in the 3' untranslated region [5,6] which may influence VDR mRNA stability. Five [7-11] of nine [7-15] studies reported higher breast cancer risk associated with the *Bsm1 bb *genotype. Other VDR 3' SNPs or haplotypes have been inconsistently related to breast cancer [16].

The presence of the *Fok1 f *allele in the 5' promoter region of the VDR (rs2228570) results in production of a VDR protein that is three amino acids longer and less effective as a transcriptional activator [16]. The *ff *genotype was associated with a statistically significant 34% higher breast cancer risk in the Nurses' Health Study [15]. Previous smaller studies of *Fok1 *and breast cancer risk were null [7,13,17].

Gene-environment interactions may explain in part the inconsistencies in the association between VDR SNPs and breast cancer across studies. Dietary factors are known to impact the vitamin D endocrine system [18], and recent studies suggest that diet may also influence autocrine/paracrine vitamin D metabolism [19]. Few studies have examined interactions between dietary factors and VDR polymorphisms in relation to breast cancer risk. Furthermore, polymorphisms in other genes in the vitamin D metabolic pathway have not been examined in relation to breast cancer risk.

We examined several polymorphisms in the VDR gene, their associated haplotypes, and polymorphisms in the vitamin D-binding (group-specific component [*GC*]) protein and 1,25(OH)_2_D degradation enzyme (*CYP24A1*) genes in relation to breast cancer risk in a nested case-control study of postmenopausal women enrolled in a large prospective cohort. Of particular *a priori *interest was whether calcium and vitamin D intakes modified associations by genotype.

## Materials and methods

### Study population

Women in this analysis were participants in the Cancer Prevention Study (CPS) II Nutrition Cohort, a prospective study of cancer incidence among men and women from 21 states in the US, established by the American Cancer Society in 1992 [20]. At enrollment, 86,404 men and 97,788 women completed a 10-page, self-administered questionnaire on medical, dietary, reproductive, and lifestyle variables. Beginning in 1997, follow-up questionnaires were sent to cohort members every 2 years to update exposure information and to ascertain newly diagnosed tumors.

From June 1998 through June 2001, blood specimens were collected from 21,965 Nutrition Cohort postmenopausal women. Using blood samples, we identified 502 breast cancer cases who were cancer-free in 1992. For each case, we randomly selected one postmenopausal female control who had provided a blood sample and was cancer-free (except non-melanoma skin cancer) at the time of the case's diagnosis [21]. Controls were individually matched to cases on birth date (± 6 months), race/ethnicity (Caucasian, African-American, Hispanic, Asian, other/unknown), and date of blood collection (± 6 months) using risk-set sampling. After excluding 2 cases with no match, 500 cases and 500 controls were available for analysis. All aspects of the CPS II cohort were approved by the Emory University (Atlanta, GA, USA) Institutional Review Board.

### Laboratory methods

Genotyping was performed at Applied Biosystems (ABI, Foster City, CA, USA) by laboratory personnel blinded to case-control status; blinded quality control samples for 10% of participants were inserted to validate genotyping procedures. TaqMan (ABI, Foster City, CA, USA) was used to distinguish alleles of the VDR gene at restriction enzyme sites *Fok1*, *Bsm1*, *Apa1*, and *Taq1*. A variable-length poly(A) sequence in the 3' untranslated region was assessed using methods previously described [6] and was classified as short (S, A_14_-A_17_) or long (L, A_18_-A_22_).

We also genotyped selected SNPs in vitamin D pathway genes that were relatively common and validated at study inception. Using TaqMan, we genotyped a T432G aspartate to glutamate SNP (rs7041) and an A436C threonine to lysine SNP (rs4588), both in exon 11 of the *GC *gene, which codes for the vitamin D-binding protein (DBP). We also measured a synonymous A→G SNP in exon 4 of the *CYP24A1 *gene (rs2296241), which encodes 24(OH)ase, which initiates degradation of 1,25(OH)_2_D.

Concordance for quality control samples was 100%. The overall success rate for genotyping assays was at least 95%, except for *Bsm1 *(93%). All SNPs were in Hardy-Weinberg equilibrium (*p *>0.05).

### Dietary assessment

Calcium, vitamin D, and dairy product intakes were measured in 1992 to 1993 [20] using a semi-quantitative 68-item food frequency questionnaire (FFQ), which is a modification of the brief 'Health Habits and History Questionnaire' developed by Block and colleagues [22]. Dietary and total nutrient intakes were estimated using the Diet Analysis System version 3.8a (Division of Cancer Prevention and Control, National Cancer Institute, Bethesda, MD, USA) [23]. Total calcium estimates (mg/day) included contributions from diet, individual calcium supplements (250, 500, 600, or 750 mg per tablet), and multivitamin pills (estimated at 130 mg per multivitamin pill). Vitamin D values were added to the nutrient database using US Department of Agriculture sources [24]. Total intake of vitamin D (IU/day) was estimated from diet (fortified milk, cereals, and fish) and multivitamin usage (400 IU per pill). Nutrient estimates were adjusted for total energy using the residual method [25].

The FFQ was validated using four random 24-hour recalls collected throughout a 1-year period as the comparison measure among 441 Nutrition Cohort participants [26]. Median energy-adjusted, attenuation-corrected Pearson validity correlations and 95% confidence intervals (CIs) among women were high for dietary calcium (*r *= 0.66, 95% CI 0.51 to 0.76) and for dairy products (*r *= 0.63, 95% CI 0.45 to 0.77).

### Statistical analyses

We used a χ^2 ^test to assess whether VDR, GC, and CYP24A1 genotype distributions were in Hardy-Weinberg equilibrium. Haplotypes of the VDR SNPs *Bsm1*,*Apa1*, and *Taq1 *and the poly(A) repeat were estimated using the expectation-maximization algorithm implemented in the TAGSNPS program [27]. To account for matching, conditional logistic regression was used to examine the association between all SNPs and the VDR haplotypes and postmenopausal breast cancer risk. We adjusted for additional risk factors and confounders as noted below. The main analysis combined invasive (*n *= 393) and *in situ *(*n *= 107) breast tumors; sensitivity analyses excluding *in situ *cases showed similar findings; thus, all cases are presented.

Covariates included education, age at menopause and menarche, parity, age at first live birth, personal history of benign breast disease, family history of breast cancer in mother or sisters, postmenopausal hormone replacement therapy use, and body mass index. Adjustment for alcohol consumption did not change the effect estimates and was not included.

We examined whether dietary or total calcium or vitamin D modified associations by genotype; interaction terms between genotypes and each dietary variable in continuous form were included in the conditional logistic regression models. Total energy intake was included in the models examining dietary interactions. Two-way interactions of the VDR haplotypes, *Fok1*, GC SNPs and the CYP24A1 SNP were examined. Statistical interaction was evaluated using the Wald test; a *p *value of less than 0.05 was considered statistically significant.

## Results

Descriptive characteristics of participants in this study are presented in Table 1. Cases and controls had a median age of 62 years (range 43 to 75 years) at enrollment in 1992, and 99% were Caucasian. None of the VDR genotypes or polymorphisms in the vitamin D-binding (*GC*) or *CYP24A1 *gene was significantly associated with overall postmenopausal breast cancer risk (Table 2). Heterozygote and homozygote variants for SNPs near the 3' end of the VDR gene were combined because of similar odds ratios (ORs). Three VDR haplotypes were identified that explained more than 96% of the variance among these SNPs: *baTL *(45.2%), *BAtS *(38.7%), and *bATL *(12.3%). Haplotype associations were similarly null: *baTL *OR = 0.89 (95% CI 0.66 to 1.20), *BAtS *OR = 1.24 (95% CI 0.92 to 1.66), and *bATL *OR = 0.96 (95% CI 0.69 to 1.32) for one or two copies versus none.

In analyses stratified by dietary factors, total calcium intake (but not vitamin D intake) modified the associations between polymorphisms near the 3' end of the VDR and breast cancer risk (Table 3). Compared to women with less than median total calcium intakes (<902 mg/day) and the *BB *or *Bb Bsm1 *genotype (reference group), women with calcium intakes of more than 902 mg/day and the *bb *genotype had a 39% lower risk of breast cancer (*p*_interaction _= 0.01). Similar findings were observed for those with the *Taq1 TT *and the poly(A) LL tail. These interactions were similar and statistically significant for dietary calcium, and no effect modification was observed with dietary vitamin D or dairy product consumption. Diet did not modify the associations between *GC *or *CYP24A1 *gene polymorphisms and breast cancer risk (not shown). We also observed no statistically significant effect modification in analyses stratified by median UV index for the state of residence as a proxy for cutaneous production of vitamin D. In addition, we observed no significant interactions between any two-way combination of VDR haplotypes, *Fok1*, or CYP24A1 or GC genotypes or in specific combinations of *Fok1 *and poly(A) genotypes.

## Discussion

None of the genotypes or haplotypes examined from the vitamin D pathway was associated with postmenopausal breast cancer risk in all women. However, women with a calcium intake higher than the median were at lower breast cancer risk if they had the VDR Bsm1 *bb*, Taq1 *TT*, or the poly(A) long repeat (LL) genotype. Among women with a calcium intake lower than the median, these VDR genotypes were not associated with lower breast cancer risk.

The lack of association between the *Bsm1 bb *genotype or associated polymorphisms in the 3' region and breast cancer risk is in contrast to findings in five studies that reported increased risk of breast cancer, mostly among Caucasian women with the *bb *genotype [7-11]. Four other studies, plus ours, have not replicated this association [12-15]; two of these were in Hispanic [13] and Taiwanese [14] women. Other SNPs in the *baT *haplotype allele likewise have been inconsistently related to breast cancer risk [16]. It is unclear whether chance or underlying differences in the populations studied explain these inconsistencies. It is conceivable that differences in background risk factor profiles (menopausal status, reproductive factors, obesity, or dietary intake) contribute to these variations. Most of these studies included both pre- and postmenopausal cases; in the one study that presented results stratified by menopausal status [15], results for the *BsmI BB *versus the *bb *allele were similar for premenopausal (OR = 0.92) and postmenopausal (OR = 0.94) women.

In our study, dietary calcium intake, but not vitamin D intake, significantly modified the association between VDR SNPs near the 3' end of the gene and breast cancer risk. Both nutrients have direct effects on cell proliferation and differentiation of several cancer cell lines *in vitro *[28,29]. In a rodent model, calcium reduces fat-induced cell proliferation by maintaining intracellular calcium concentrations [30]. It is plausible that polymorphisms in the VDR gene could modify the effects of exposures such as vitamin D or calcium on breast cancer risk, although the specific mechanisms in humans are unknown. Although vitamin D intake did not modify risk in our analysis, we did not measure plasma 25(OH)D, an integrated measure of vitamin D status from diet and UVB synthesis. Higher circulating levels of the storage form of vitamin D (25(OH)D) may lower breast cancer risk [9,31], potentially by providing substrate for breast tissue-specific synthesis of 1,25(OH)_2_D [3]. In one case-control study in the UK, women with low plasma 25(OH)D levels and the *BsmI bb *VDR genotype had a 6.8-fold higher risk of breast cancer than individuals with higher 25(OH)D levels and the *BB *or *Bb *genotype [9]. Thus, calcium or related nutrients may influence tissue-specific vitamin D metabolism in ways that are as yet unclear, possibly varying by VDR genotype. The interactions we observed may also be due to chance and should be replicated.

Our null findings for the *Fok1 ff *SNP and breast cancer risk do not replicate the positive association observed in the Nurses' Health Study [15] but do agree with those of three smaller studies [7,13,17], nor did we replicate previously reported potential interactions between different VDR polymorphisms observed by others [8,15]. Guy and coll*eagues *[8] reported an approximately two-fold increase in risk among women with the FFLL or FfLL genotypes versus other combinations; among patients with metastases, the risk was three-fold greater. The mix of grade, lymph node involvement, or metastases in cases studied may also contribute to variability in findings across studies.

We observed no association with two non-synonymous polymorphisms in the *GC *gene (codes for DBP) or one in *CYP24A1 *and postmenopausal breast cancer. The polymorphic DBP facilitates vitamin D actions, and DBP alleles differ in their affinity for 1,25(OH)_2 _D [32]. The *CYP24A1 *gene codes for 24(OH)ase, which degrades 1,25(OH)_2_D. The potential for the SNPs we examined to alter DBP or CYP24A1 function is possible but has not been studied. Some studies have suggested a relationship of these SNPs with other outcomes, including asthma, glucose tolerance, and bone mineral density [33-35]. We are not aware of any studies that examined these SNPs in relation to breast cancer.

Limitations of this analysis include the use of a single assessment of diet, on average 6 years before diagnosis of breast cancer, which may have led to misclassification of dietary intake. We did not measure blood levels of 25(OH)D to assess vitamin D status. However, our FFQ was validated [26] and diet was assessed before cancer diagnosis. Larger case numbers would have provided greater statistical power to examine gene-gene and gene-diet interactions; however, this study is among the largest published to date for postmenopausal breast cancer.

## Conclusion

Our research does not support an independent association between selected polymorphisms in the VDR, *GC*, or *CYP24A1 *genes and postmenopausal breast cancer risk among US Caucasian women. However, calcium intake appeared to modify the association of VDR SNPs with breast cancer risk. More research is needed to determine whether environmental factors, particularly diet, influence cancer risk by vitamin D pathway genotype.

## Abbreviations

CI = confidence interval; CPS = Cancer Prevention Study; DBP = vitamin D-binding protein; FFQ = food frequency questionnaire; GC = group-specific component; OR = odds ratio; SNP = single-nucleotide polymorphism; VDR = vitamin D receptor.

## Competing interests

The authors declare that they have no competing interests.

## Authors' contributions

MLM conceived the study and drafted the manuscript. VLS, HSF, EEC, and RMB made substantial contributions to the conception and design of the study. MJT, CR and EEC made substantial contributions to the acquisition and interpretation of data. WRD conducted the statistical analyses. All authors were involved in revising the manuscript critically for important intellectual content. All authors read and approved the final manuscript.

## Figures and Tables

**Table 1:** Selected characteristics of breast cancer cases and controls

Variable	Cases *n *= 500	Controls *n *= 500
	Mean ± standard deviation	
Age at baseline (years)	61.9 ± 6.0	61.9 ± 6.0
Age at menarche (years)	12.7 ± 1.4	12.6 ± 1.4
Age at menopause (years)	48.5 ± 6.4	48.1 ± 6.0
Parity^a^	3.0 ± 1.2	3.3 ± 1.5
Race	Percentage	
White	98.6	98.8
Other or missing	1.4	1.2
History of breast cyst	38.8	30.0
Family history of breast cancer	20.2	15.0
Ever pregnant	88.8	90.0

^a^Mean number of children only among parous women.

**Table 2:** Associations between SNPs in VDR, GC, and CYP24A1 genes and postmenopausal breast cancer risk^a^

	Frequency		Matched only		Multivariate-adjusted^b^	
SNP	Cases	Controls (%)	OR	95% CI	OR	95% CI
VDR						
Fok1 (rs2228570)						
FF	191	185 (37.0)	1.00	-	1.00	-
Ff	207	227 (45.4)	0.89	(0.68–1.17)	0.78	(0.58–1.05)
ff	77	68 (13.6)	1.11	(0.75–1.63)	0.98	(0.65–1.50)
Taq1 (rs731236)						
tt or Tt	336	312 (62.4)	1.00	-	1.00	-
TT	158	180 (36.0)	0.82	(0.63–1.06)	0.83	(0.62–1.12)
Apa1 (rs7975232)						
AA	147	129 (25.8)	1.00	-	1.00	-
Aa or aa	341	348 (69.6)	0.87	(0.65–1.15)	0.87	(0.64–1.18)
Bsm1 (rs1544410)						
BB or Bb	321	290 (58.0)	1.00	-	1.00	-
bb	151	170 (34.0)	0.81	(0.61–1.06)	0.84	(0.62–1.15)
Poly(A) tail						
SS or SL	328	304 (60.8)	1.00	-	1.00	-
LL	158	177 (35.4)	0.83	(0.63–1.08)	0.83	(0.61–1.12)
GC protein (rs4588)						
C/C	240	246 (51.7)	1.00	-	1.00	-
A/C	202	186 (39.1)	1.11	(0.84–1.45)	1.11	(0.82–1.48)
A/A	48	44 (9.2)	1.13	(0.73–1.76)	1.30	(0.80–2.13)
GC protein (rs7041)						
G/G	154	149 (30.4)	1.00	-	1.00	-
G/T	237	235 (48.0)	0.98	(0.72–1.33)	0.94	(0.67–1.31)
T/T	103	106 (21.6)	0.94	(0.66–1.33)	0.96	(0.65–1.41)
CYP24A1 (rs2296241)						
A/A	141	139 (28.4)	1.00	-	1.00	-
A/G	254	253 (51.6)	0.99	(0.74–1.31)	1.03	(0.75–1.41)
G/G	99	98 (20.0)	1.00	(0.70–1.43)	1.05	(0.71–1.56)

^a^All models contain a variable for unknown SNP genotype which is not presented. ^b^Models are adjusted for body mass index, history of breast cyst, hormone replacement therapy use, family history of breast cancer, education, parity and age at first birth combined, age at menarche, and age at menopause. CI, confidence interval; GC, group-specific component; OR, odds ratio; SNP, single-nucleotide polymorphism; VDR, vitamin D receptor.

**Table 3:** VDR genotype and postmenopausal breast cancer risk stratified by total calcium and vitamin D intakes^a^

	Total calcium (mg/day)				Total vitamin D (IU/day)			
	<902		902+		<222		222+	
SNP	OR	95% CI	OR	95% CI	OR	95% CI	OR	95% CI
Fok 1								
FF	1.00	-	0.91	(0.56–1.47)	1.00	-	0.89	(0.55–1.45)
Ff	0.79	(0.50–1.25)	0.80	(0.50–1.26)	0.72	(0.45–1.14)	0.88	(0.56–1.39)
ff	1.08	(0.56–2.09)	0.63	(0.32–1.24)	1.03	(0.53–1.99)	0.68	(0.36–1.29)
	*p*_interaction _= 0.34^b^				*p*_interaction _= 0.18			
Taq1								
tt or Tt	1.00	-	1.16	(0.81–1.65)	1.00	-	1.02	(0.70–1.49)
TT	1.24	(0.79–1.94)	0.64	(0.41–0.99)	0.88	(0.56–1.38)	0.77	(0.50–1.21)
	*p*_interaction _= 0.01				*p*_interaction _= 0.36			
Apa1								
AA	1.00	-	0.86	(0.49–1.50)	1.00	-	0.86	(0.48–1.53)
Aa or aa	0.86	(0.53–1.38)	0.74	(0.47–1.17)	0.77	(0.49–1.20)	0.82	(0.52–1.31)
	*p*_interaction _= 0.77				*p*_interaction _= 0.47			
Bsm1								
BB or Bb	1.00	-	1.05	(0.71–1.55)	1.00	-	0.90	(0.60–1.35)
bb	1.20	(0.73–1.95)	0.61	(0.38–0.96)	0.72	(0.44–1.18)	0.78	(0.49–1.26)
	*p*_interaction _= 0.01				*p*_interaction _= 0.90			
Poly(A) tail								
SS or SL	1.00	-	1.16	(0.81–1.67)	1.00	-	0.96	(0.66–1.41)
LL	1.18	(0.74–1.87)	0.66	(0.42–1.04)	0.79	(0.50–1.26)	0.77	(0.49–1.21)
	*p*_interaction _= 0.02				*p*_interaction _= 0.72			

^a^Multivariate adjusted models contain the SNP, dietary exposure, and interaction term and adjust for body mass index, history of breast cyst, hormone replacement therapy use, family history of breast cancer, education, parity and age at first birth combined, age at menarche, age at menopause, and total energy intake. ^b^Interaction terms were created using continuous form of dietary variables. CI, confidence interval; OR, odds ratio; SNP, single-nucleotide polymorphism; VDR, vitamin D receptor.
